# An Efficient Routing Protocol Based on Stretched Holding Time Difference for Underwater Wireless Sensor Networks

**DOI:** 10.3390/s19245557

**Published:** 2019-12-16

**Authors:** Zahid Wadud, Khadem Ullah, Abdul Baseer Qazi, Sadeeq Jan, Farrukh Aslam Khan, Nasru Minallah

**Affiliations:** 1 Department of Computer Systems Engineering, University of Engineering and Technology Peshawar, Peshawar 25000, Pakistan; zahidmufti@nwfpuet.edu.pk (Z.W.); n.minallah@uetpeshawar.edu.pk (N.M.); 2 National Center of Big Data and Cloud Computing (NCBC) UETP, University of Engineering and Technology Peshawar, Peshawar 25000, Pakistan; 14pwcse1224@uetpeshawar.edu.pk; 3 Department of Software Engineering, Bahria University, Islamabad 44000, Pakistan; abq.buic@bahria.edu.pk; 4 National Center for Cyber Security-UETP, University of Engineering and Technology Peshawar, Peshawar 25000, Pakistan; sadeeqjan@uetpeshawar.edu.pk; 5 Center of Excellence in Information Assurance, King Saud University, Riyadh 11653, Saudi Arabia

**Keywords:** Underwater Wireless Sensor Networks (UWSNs), Extended Energy-Scaled and Expanded Vector-Based Forwarding (EESEVBF), Potential Forwarding Nodes (PFNs)

## Abstract

Underwater Wireless Sensors Networks (UWSNs) use acoustic waves as a communication medium because of the high attenuation to radio and optical waves underwater. However, acoustic signals lack propagation speed as compared to radio or optical waves. In addition, the UWSNs also pose various intrinsic challenges, i.e., frequent node mobility with water currents, high error rate, low bandwidth, long delays, and energy scarcity. Various UWSN routing protocols have been proposed to overcome the above-mentioned challenges. Vector-based routing protocols confine the communication within a virtual pipeline for the sake of directionality and define a fixed pipeline radius between the source node and the centerline station. Energy-Scaled and Expanded Vector-Based Forwarding (ESEVBF) protocol limits the number of duplicate packets by expanding the holding time according to the propagation delay, and thus reduces the energy consumption via the remaining energy of Potential Forwarding Nodes (PFNs) at the first hop. The holding time mechanism of ESEVBF is restricted only to the first-hop PFNs of the source node. The protocol fails when there is a void or energy hole at the second hop, affecting the reliability of the system. Our proposed protocol, Extended Energy-Scaled and Expanded Vector-Based Forwarding Protocol (EESEVBF), exploits the holding time mechanism to suppress duplicate packets. Moreover, the proposed protocol tackles the hidden terminal problem due to which a reasonable reduction in duplicate packets initiated by the reproducing nodes occurs. The holding time is calculated based on the following four parameters: (i) the distance from the boundary of the transmission area relative to the PFNs’ inverse energy at the 1st and 2nd hop, (ii) distance from the virtual pipeline, (iii) distance from the source to the PFN at the second hop, and (iv) distance from the first-hop PFN to its destination. Therefore, the proposed protocol stretches the holding time difference based on two hops, resulting in lower energy consumption, decreased end-to-end delay, and increased packet delivery ratio. The simulation results demonstrate that compared to ESEVBF, our proposed protocol EESEVBF experiences 20.2% lesser delay, approximately 6.66% more energy efficiency, and a further 11.26% reduction in generating redundant packets.

## 1. Introduction

Smart coasts (SCs) are rapidly gaining importance as a major contributing factor to sustainable communities [1]. Key features of SCs are the monitoring of water quality, water pollution, seismic activity, eco-system, and the overall management of the coastal zones. To effectively implement these features, uninterrupted collection, monitoring, detection, and management of various aquatic parameters are required. Such continuous monitoring of oceanographic parameters is only made possible by employing Underwater Wireless Sensor Networks (UWSNs) [2], which have become the technological underpinning of SCs.

Several sensor nodes, operated by battery, are deployed in water-based environments to form UWSNs. Such nodes are equipped with sensing, communicating, and storing capabilities of various physical aquatic parameters. A sink floating at the surface of the sea receives the sensed data and further forwards it to the onshore monitoring station. Each communication approach is marred by unique challenges when employed in the underwater environment [3]. The communication among sensor nodes may be carried out through optical waves [4,5], electromagnetic waves [6,7], or acoustic waves [8].

Unlike underwater communication, the preferred choice for terrestrial communication has been electromagnetic signals at radio frequencies because of their wider bandwidth, lower energy consumption per bit, and smaller propagation delays. Therefore, communication from the floating sinks to the onshore monitoring stations employ this mode. On the other hand, high conductivity of seawater leads to severe attenuation and significant absorption losses, making it a bad choice for underwater communications. Similarly, sightline is a requirement in optical communications between parties and is not always possible. Moreover, the distance over which optical communication can successfully take place is drastically curtailed by water turbidity, making it unviable for underwater communications.

The modality of choice, and hence the most widely used one for underwater networks is the acoustic communication, where the network is also termed as underwater acoustic sensor network (UASN). UASNs also face various challenges such as fading, resulting in high error rates and bandwidth limitations due to multipath [9]. Moreover, acoustic signals in an aquatic environment travel much slower compared to radio signals in a terrestrial environment. These challenges result in limitations in synchronization, data routing, and information regarding the network state. This explains why traditional communication approaches, otherwise successful for terrestrial sensor networks, cannot be effectively used in the underwater environment.

Most of the challenges faced by UASNs listed above are interdependent, making it even more complex to design optimal solutions. In vector-based routing protocols, each node calculates the holding time based on the node/network parameters, e.g., the distance to sink, middle of the virtual pipeline, and receiving node distance from the previous hop’s sender. Holding time is only estimated after a node first checks and ensures that it is located within the virtual pipeline. This procedure takes place upon receiving the first duplicate of a packet from downstream nodes. The timer is set up based on the (estimated) holding time. When the timer expires, the node forwards the packet, if no other copy from its neighbors is received. At the same time, the remaining nodes will suppress their packet forwarding protocols in favor of the node, which has the minimum holding time in all the surrounding nodes.

For faster propagation, such protocols (vector-based) calculate the holding time based on the nodes’ proximity. In addition, the proximity of the middle of the pipeline and the distance to the sink are also considered for estimation. End-to-end delay reductions occur while packets are forwarded through these nodes; however, it causes energy depletion, resulting in a void energy hole problem. For this reason, it is required to have a fair energy balancing among the nodes inside the vector and the whole network. Therefore, holding time estimations consider the energy factor to increase the network lifetime. However, the shortest path between the sender and the sink does not guarantee fair distribution of load/energy in the network. Alternatively, better decisions regarding forwarding based on precise holding time estimations are achieved when each node in the network is updated with the network state information (complete or partial network). Exchange of control packets makes this network state information available; however, this traffic puts additional burden on bandwidth, energy consumption and error rates. Therefore, suggested forwarding schemes for UASNs should address these limitations and offer a balanced tradeoff. Further improvement to reduce the number of duplicate packets can be made if the immediate neighbors estimate their holding times to be greater than the propagation delays.

Higher energy consumption, channel errors and long propagation delays of acoustic signals are well-established challenges in aquatic environments [10]. Energy consumption and propagation delays are phenomenally increased when the far away node of the network sends packets to the sink node (fixed at a particular point). For efficient collection of data, sink mobility has been suggested by various schemes in the literature. Mobile Sinks (mobile stations) are nodes that can change their position in two ways: (1) autonomously, and (2) via anchored ropes/vessels. An example of autonomously changing positions is the autonomous underwater vehicles (AUVs) [11]. The mobile sink is assumed to have frequent refueling and recharging made available to ensure roaming in the network. Therefore, the proposed routing schemes for UASNs may also consider the effectiveness of using a mobile sink.

In this paper, Extended Energy-Scaled and Expanded Vector-Based Forwarding (EESEVBF) protocol exploits the holding time mechanism to suppress duplicate packets. Moreover, the proposed protocol also tackles the hidden terminal problem due to which a reasonable reduction in duplicate packets initiated by the reproducing nodes occurs. The holding time is calculated based on the following four parameters: (i) the distance from the boundary of the transmission area relative to the PFNs’ inverse energy at the 1st and 2nd hop, (ii) distance from the virtual pipeline, (iii) distance from the source to the PFN at the second hop, and (iv) distance from the first-hop PFN to its destination. Therefore, the proposed protocol stretches the holding time difference based on two hops, resulting in lower energy consumption, decreased end-to-end delay, and an increased packet delivery ratio.

### Motivation and Contributions

The vector-based routing protocols use pipeline for directionality purposes along with the positional information of the node for calculating the holding time. The proximity of the node towards the destination point (upward packet advancement) reduces the end-to-end delay and considering the normalized energy of the PFNs from the first and second hop, reduces the energy consumption in the network. Based on these factors, we propose Extended Energy-Scaled and Expanded Vector-Based Forwarding (EESEVBF) protocol. EESEVBF considers the following points in designing the holding time mechanism for the PFNs:

1. To avoid the void hole problem, the holding time of the PFNs is calculated based on the energy of the neighboring nodes. The holding time between two PFNs of a source node depends on the propagation distance between them, i.e., a larger holding time difference reduces duplicate packets, resulting in reduced overhead in the network. A small variation in the energy levels of neighboring nodes can affect the difference between the holding time of the nodes.
2. The nodes are prioritized on their position with respect to the virtual pipeline. The distance between the sender and the forwarder is added in the holding time mechanism, which helps in minimizing the end-to-end delay. This factor generally contributes to the movement of packets by a large distance in a specific direction towards the sink.
3. The nodes manipulate the timer information of their common neighbors with the source node, modeling the network as a real time system. The node with the minimum holding time in all the PFNs of a source node forwards the packet within a very short time. The packet is further forwarded by the nodes in the second hop using the same mechanism.
4. Energy balancing is accomplished by using the residual normalized energy information of the candidate nodes in the estimation of the holding time. This process aims to have the same energy level of all nodes within the transmission range of the source node. Therefore, no single node will go to the dead state alone.
5. The upward packet advancement is effectuated by taking into consideration the depth information of the first two hops from the current source node. The forwarder nodes are prioritized resulting in more advancement in the first two hops of the source node compared to the subsequent ones.
6. Further minimization in the energy tax is achieved by suppressing the packet forwarding initiated from the reproducing regions. The proposed protocol uses control (announcement) packets for suppressing the duplicate packets from regions where the forwarder nodes have no access.
7. Energy optimization is achieved by exploring the sending and receiving energy in the whole network life with the linear programming technique.

The novelty of our approach is given in the following points:

- The best route forwarder is decided based on the holding time value calculation, which also considers the second hop.
- The timer value of the first hop is not affected but only the priority of transmission is changed based on their succeeding hops.
- In addition to the first hop, the second hop is also used in the calculation of all the three parameters (Energy factor (Ef), Positioning factor(Pf) and Cultivate Packet Advancement).
- We introduce a novel concept of control packets for addressing the hidden terminal problem.

The simulation results manifest that EESEVBF reduces the energy tax, lessens the end-to-end delay, and ensures the reliability compared to ESEVBF.

This paper is further organized as follows: Section 2 reviews the previous work related to the proposed EESEVBF. Section 3 describes the problem statement and background. Section 4 details the architecture of our proposed protocol and the holding time value calculation. The experimental setup is described in Section 5. In Section 6, we present and discuss the simulation results. Finally, the conclusions and future directions are given in Section 7.

## 2. Related Work

Several studies have already been carried out for efficient routing protocols in UWSNs [12,13,14]; however, we describe the closely related work in this section, i.e., all those protocols that depend on the pipeline radius for directionality purposes using a node’s relative coordinates [15] and timer information [16] for broadcasting.

In [17], the authors propose Vector-Based Forwarding (VBF), which draws a fixed virtual pipeline between source and terminus point for forwarding a data packet. The forwarding decision can be made by considering the relative position of the node with reference to the pipeline. Upon reception of a data packet by a sensor node, it verifies if it is inside the virtual pipeline. If the answer is yes, then it computes the *desirableness factor* (α). The *desirableness factor* is the ratio between the virtual pipeline width to the sum of the distance of the forwarder from the center and the source node. The forwarder nodes that are closer to the pipeline are selected each time, resulting in an energy hole in a short period of time. When the number of nodes in the network increases, it also increases the duplicate copies of the data packet due to the lesser difference between the holding time as compared to the propagation delay. Accordingly, more energy consumption occurs throughout the network and most of the packets do not reach their destination. When the network density is lower, finding a suitable node becomes more challenging. For an efficient packet delivery ratio, a single path between the terminus point and the forwarder must remain, which is indeed inflexible in VBF.

To overcome the shortcomings in VBF, researchers proposed hop-by-hop VBF (HH-VBF)  [18]. The HH-VBF defines a virtual pipeline on each successive forwarding hop, instead of using a single pipeline. Moreover, it improves reliability by considering pipelines to find more suitable forwarding nodes. The node transmission range is similar to that of the pipeline radius. The mechanism for calculating the timer value is similar to that of VBF. Because of the reduction in the number of void holes, the PDR is improved in HH-VBF compared to its predecessor VBF. Similar to the VBF protocol, HH-VBF can not also apply fair energy to the nodes in the network. The inadequacies of HH-VBF are covered by Adaptive HH-VBF (AHH-VBF) [19]. AHH-VBF dynamically alters the transmission power and forwarding zones. The transmission power for each forwarding data packet is enumerated to the farthest node in the transmission region and the forwarding zone is defined based on first-hop qualified nodes’ density at each hop. The motivation behind the different values of the pipeline radius is to reduce the broadcasting to an acceptable level. AHH-VBF uses the *controlled packets* to attain forwarding zone and transmission power adaptiveness. The *request packet* is sent by the source multiple times at different transmission powers and it retains the neighbors table, while in response, it receives the *acknowledgment packet*. In this process, if the total nodes are fewer than the predefined threshold value, then the maximal power level is selected, otherwise the power level is adjusted appropriately. Adjusting the transmission power and the pipeline radius results in efficient energy tax. However, each time the same set PFNs will always be selected, which violates the fair distribution of energy among the nodes. Moreover, the dynamic power does not ensure the prevention of duplicate packets as well as the selection of qualified forwarders.

Let us now consider those protocols that do not use holding time calculations, but they are based on location. The authors in [20] propose Focused Beam Routing (FBR) protocol, which uses the concept of directional power alteration in seeking directionality. Prior to forwarding packets, the source node gradually increases the flooding angle and power based on gradients defined in advance. In FBR, the source node sends many *Request To Send* (*RTS*) packets in each direction and needs to wait for the *Clear To Send* (*CTS*) packet. Many *RTS* packets need to be sent by the node and then it must wait for receiving the *CTS* packets. These *CTS* packets are expected from the neighbors in the direction of the beam. The control overhead packets can incur higher costs due to higher energy consumption and end-to-end delays at each hop in a crowded network. In FBR, the delay is caused due to the exchange of control packets (*RTS* and *CTS*), which lead the authors in  [21] to introduce Layer by Layer Angle-Based Flooding (L2-ABF) protocol. A cone-shaped angle of flooding is created to the shallower layer facing the terminus point. In L2-ABF, the angle of transmitting power (i.e., cone’s length/width) is based on the Euclidean distance and the packet’s speed between the sender and receiver nodes. The scheme sends multiple duplicate packets in dense and random network scenarios.

The authors in [22] proposed Directional Flooding-based Routing (DFR), which is a receiver and location-based scheme. The nodes in the network can find their relative coordinates, qualified node and the destination sink location. In DFR, all the nodes do not need to calculate the holding time value for their timer because of directly transmitting mechanism in an upward direction. Moreover, the broadcasting of data packets is confined within a certain range to improve the energy efficiency. The width of the flooding is dynamically traded off with signal strength. The energy consumption sometimes leads to its maximum due to the unnecessary width of the power flooding, which alternatively reduces the packet delivery ratio.

A modified Dynamic Source Routing-based Location-Aware Source Routing (LASR) has been proposed in [23]. It uses link quality, i.e., expected transmission count (ETX) and location awareness as routing metrics to forward packets towards the sink node. As it uses source routing, therefore, the packet size is directly proportional to the number of hops that the packet has been relayed. Furthermore, it requires flooding of the route request in the entire network to find a suitable route towards the destination, which drastically reduces the network performance and consumes network resources.

To minimize energy tax per node, increase packet delivery ratio, and reduce end-to-end delay, the literature focuses on different sink mobility scenarios which are briefly discussed below.

Instead of covering the whole network, authors in [24] proposed autonomous underwater vehicle (AUV) to collect data from path nodes (selected nodes). In AUV, the path nodes act like local data collection points, avoiding the sink to traverse the whole network and receiving data efficiently with less energy through already well-defined paths.

Another relevant protocol is the Aided Underwater Routing Protocol (AURP) [25], which receives packets from multiple gateway nodes. The scheme uses different (heterogeneous) channels to collect data to minimize and control sink mobility. The addition of heterogeneous channel enables AURP to use either lower bit rate with long-range channel or higher bit rate with short-range channel.

In [26], the authors proposed mobile sink mobicast or geocast in the Euclidean three dimensions for the underwater sensor network (USN). The scheme mainly focuses on reducing the void hole occurrence due to the energy tax throughout the network when the mobile sink collects data. The ZOR (zone of reference) is achieved by dividing the whole geographic 3D USN zone into multiple sub-zones. The sink receives the data from nodes within ZOR and traverses the trajectory through the well-defined paths created in the initializing phase.

In [27], the authors propose a new protocol called DOlphin and Whale Pods Routing (DOW-PR) protocol consisting of two parts i.e., dolphin and the further improved version, whale pods. The scheme enhances the reliability, end-to-end delay and minimizes the energy consumption compared to Weighting Depth and Forwarding Area Division (WDFAD) routing protocol. The proposed scheme selects the path where traffic congestion is minimum, which results in initiating the least number of duplicate packets. The counterpart whale pods embed sink in the middle in a specific region, which gives a very high data rate and prolongs the network lifetime.

Sendra et al. [28] performed electromagnetic/radio communication in UWSN using a frequency band of 2.4 GHz. The behavior of radio signals was analyzed as a function of network parameters, i.e., data rates, working frequency and modulations. The proposed protocol focused on rectifying the existing models of electromagnetic communication in freshwater and compared it with analytical models in the specified environment. The Round-Trip Time (RTT) of a packet was analyzed with various modulation techniques, frequency and as a distance function between the sender and the receiver.

Sensor nodes operated by battery are self-powered. The battery of the node discharges with time, reducing the network lifetime. There exist various techniques to prolong the network lifetime. The energy consumption can be decreased if idle/sleep state is used by the sensor nodes when no data is transmitted. During this idle state, the sensor nodes operate in very low energy consumption mode. Such a transition between active and idle states requires that nodes in the network are operated at the same clock reference. For this purpose, the authors in [29] proposed Acoustic Triggered Wake-Up (AT-WUP) system embedded within a sensor architecture that can switch between active and sleep states to improve the network lifetime. Such an architecture requires only 8.71 μW energy consumption. Furthermore, the sensor nodes at idle state cause a minimum discharge (i.e., less than 1 μA), which can be easily handled by the proposed AT-WUP system. The proposed system attempts to address the issue of global clock synchronization by providing a wake-up solution for low-power nodes.

In [30], the authors proposed a protocol for delay tolerant underwater networks. The protocol uses a timer countdown interval mechanism to overcome the packet collision at the next node from the previous two nodes. Packet loss is reduced by implementing a queue to store the packet for some time in case it does not receive the expected qualified hop. Hop count information is exchanged between nodes with control packets (HELLO message). Such a HELLO message consists of the information to identify the sensor node and the distance to the destination. The transmission of such packets happens at regular intervals from the nodes with a state table with some information. These tables are to update other nodes’ tables that can initiate the transmission of other data packets. Each table is updated upon reception of a HELLO message with the information of the hop count of the sender to the sink node. This process of updating state tables is followed for all nodes. During this process, if another node is found closer to the sink that receives a HELLO message, then it starts sending the data packet (if any). On the other hand, if no node is found closer to the sink, then the HELLO message is sent to update other nodes’ tables.

Underwater Delay Tolerant Network with Probabilistic spraying (UDTN-Prob) [31] is a replication-based routing protocol that estimates the Cumulative Distributive Function (CDF) between the current node and the sink node to give priority of transmission to a node. CDF is the probability that a packet will reach its final destination by selecting the current forwarder.

In contrast to these approaches of DTN, our proposed protocol calculates the transmission priority based on the holding time ($HT_{1st}$) of the current forwarder and the minimum among the succeeding PFNs of that forwarder at the second hop ($HT_{2nd}$).

## 3. Problem Statement

**Figure 1:**
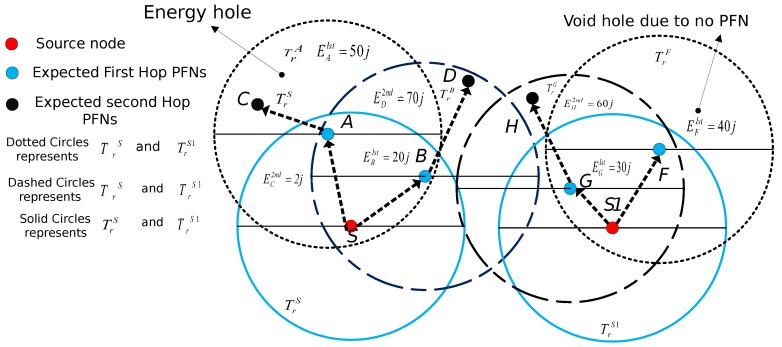
Forwarder Node Selection Scenario.

The Energy-Scaled and Expanded Vector-Based Forwarding (ESEVBF) protocol  [32] expands the holding time of AHH-VBF to suppress duplicate packet generation due to a very small difference between the holding time of the neighbor nodes as compared to the propagation delay. The propagation delay is very high in the harsh underwater environment. Upon generation/reception of a packet from its predecessor, the node forwards the packet to other nodes in its transmission range, which will result in generating a large number of duplicate packets and in more energy consumption if all nodes take part in forwarding. Therefore, it is very challenging to reduce the forwarding nodes to an acceptable level. Till now, all the protocols use the holding time mechanism to suppress duplicate packets. The holding time is calculated in ESEVBF based on: (1) the distance of the PFN from the edge of the transmission range scaled with inverse normalized energy, (2) the distance from the virtual pipeline, and (3) the distance from the sink node of that corresponding node. ESEVBF covers all the factors related to the holding time but it is based only on the first hop. In ESEVBF, the problem may arise due to the presence of void/energy hole found by the PFN of the sender. The proposed scheme will extend the holding time scenario to the second hop. In that case, there will be a higher probability to advance the packet further in the overall distance covered by the two hops from the source node. The proposed system tends to find the more suitable node, which is best with respect to the first hop as well as the second hop. The whole scenario of selecting the forwarder node is depicted in Figure 1. The small red circles represent the source nodes (S and S1), the blue circles represent the Expected First-Hop PFNs and their energies, while the black ones represent the Expected Second Hop PFNs and their energies. Similarly, there are three types of big circles in the figure representing various transmission ranges, i.e., the circles with the solid line boundary represent the transmission ranges of the source nodes, the dashed circles represent the transmission ranges at the first hop, while the dotted ones represent the transmission ranges at the second hop. As depicted in the figure, node S is the source node because it broadcasts a packet and nodes A/B are the receivers of the packet. ESEVBF selects node A for forwarding the received packet, which means that Node A will transmit the packet early in such a way that node B receives it before its own broadcasting. When Node B receives the packet transmitted by A as a duplicate packet, it suppresses both the packets (the original packet received from Source node S as well as the duplicate packet received from node A). The PFN (node C) of node A does not have sufficient energy to continue broadcasting. The proposed system will select node B for forwarding as it has a second hop PFN, which is more favorable with respect to energy and packet advancement. Therefore, the proposed protocol tends to increase the net packet advancement and finds the best favorable routing path with respect to the residual energy.

In the second scenario, as shown in the same Figure 1, node S1 has G and F PFNs in its transmission range. When node S1 broadcasts the received/generated packet, nodes F and G receive the packet, and the next forwarder is selected based on the holding time. ESEVBF selects node F as a next forwarder, which introduces the problem of void holes. In contrast, EESEVBF will give preference to node G because there is node H at the second hop, which can further continue the packet transmission process for reducing data loss.

**Figure 2:**
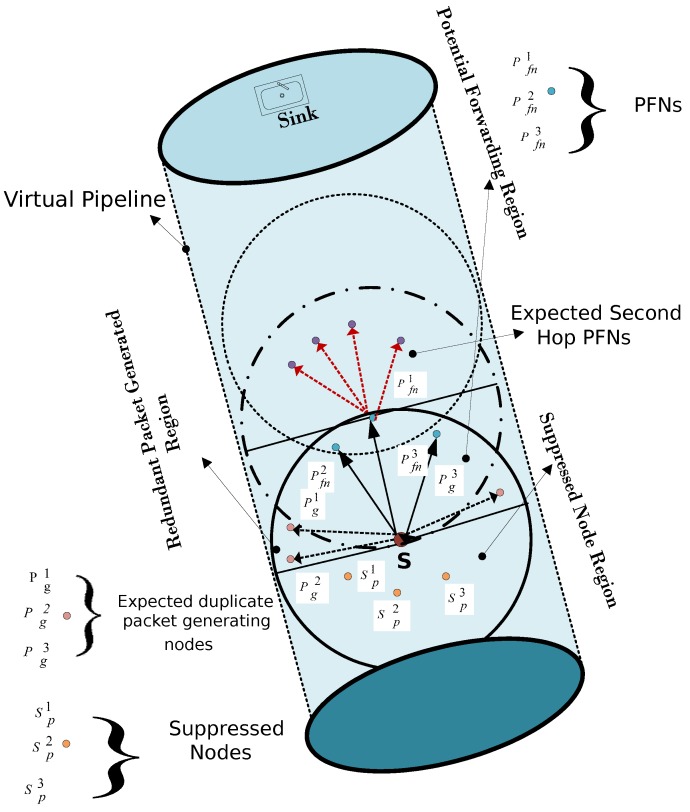
Hidden Terminal Problem Scenario.

The second problem faced in ESEVBF is the hidden terminal problem, as depicted in Figure 2. The source node broadcasts while its PFNs receive the packet. The qualified nodes are prioritized according to the holding time value. The problem occurs in a scenario when the forwarder (the node with higher priority) does the broadcasting of the packet in its own transmission zone after the expiry of the timer value, while some PFNs of the source node are not in the range (located in reproducing regions, as shown in Figure 2), and hence do not receive a copy of the original packet. This results in the generation of duplicate packets. The source node S broadcasts the packet, which is received by the nodes $P_{fn}^{1}$, $P_{fn}^{2}$, $P_{fn}^{3}$, $S_{p}^{1}$, $S_{p}^{2}$, $S_{p}^{3}$,$P_{g}^{1}$, $P_{g}^{2}$, and $P_{g}^{3}$, as shown in Figure 2. The suppressed nodes due to being deeper than the source node, directly drop the packet but $P_{fn}^{1}$, $P_{fn}^{2}$, $P_{fn}^{3}$, $P_{g}^{1}$, $P_{g}^{2}$, and $P_{g}^{3}$ calculate the holding time value and set the timers. Based on the parameters of the holding time, the highest priority node should be node $P_{fn}^{1}$. The nodes $P_{g}^{1}$, $P_{g}^{2}$ and $P_{g}^{3}$ are out of the transmission range of the highest priority node ($P_{fn}^{1}$). Therefore, they will generate duplicate packets and transmit these packets, resulting in higher energy consumption. On the contrary, EESEVBF uses control packets to suppress the reproducing nodes, as discussed below.

**Figure 3:**
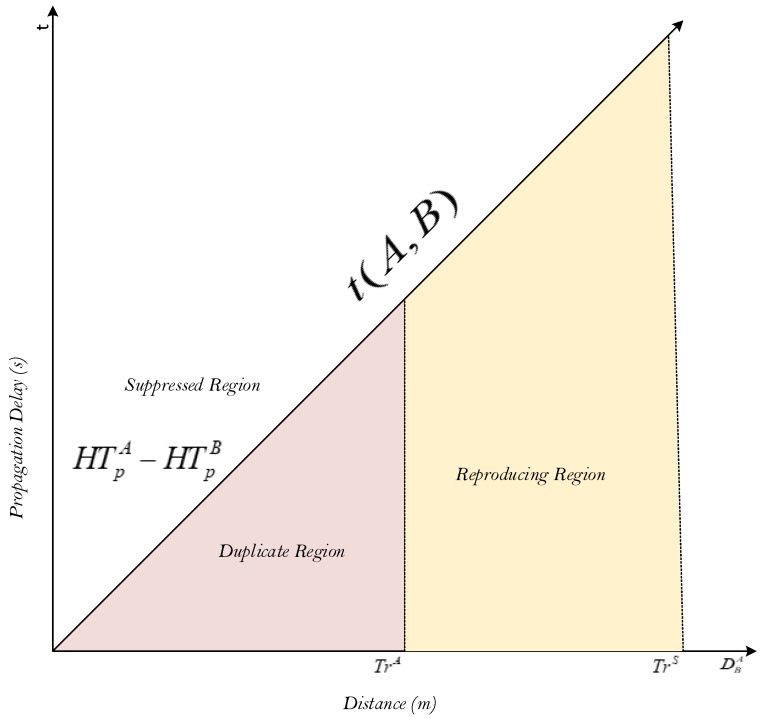
Holding time difference relationship with propagation delay.

The propagation delay τ(n1,n2) between two nodes in UWSNs is proportional to the separation distance between them, as shown in Figure 3. Node A can suppress the broadcasting of node B when the holding time difference between them is greater than the propagation delay and in that case, the suppressed otherwise duplicate region will be achieved. The reproducing region is achieved due to the hidden terminal problem. In ESEVBF, duplicate packets are initiated and forwarded in both suppressed and reproducing regions, while in EESEVBF, this is achieved by using the control packets. Mathematically, the suppressed region, duplicate region and reproducing region can be expressed in Equations (1)–(3) respectively:

(1) $$\begin{array}{cc} \; & HT_{p}^{n1}-HT_{p}^{n2}>\tau \left(n1,n2\right)\wedge Tr_{n1}\geq D_{n1}^{n2}<Tr_{S} \end{array}$$

(2) $$\begin{array}{cc} \; & HT_{p}^{n1}-HT_{p}^{n2}<\tau \left(n1,n2\right)\wedge Tr_{n1}\geq D_{n1}^{n2}<Tr_{S} \end{array}$$

(3) $$\begin{array}{cc} \; & HT_{p}^{n1}-HT_{p}^{n2}>\tau \left(n1,n2\right)\wedge Tr_{n1}\leq D_{n1}^{n2}<Tr_{S} \end{array}$$

The duplicate region can be easily avoided if the holding time difference is larger, but at the same time, it can generate a long end-to-end delay. ESEVBF estimates the holding time in such a way that the suppression is achieved frequently with the tradeoff of end-to-end delay.

### Preliminaries

The proposed EESEVBF scheme uses the following notations:

*Sink Node $S_{k}$:* Sink node in UWSN is a superficial node that is placed at the sea surface to collect data from the deployed nodes inside the water. A radio link is used by the sink nodes for communication. The packet received by the sink node is considered to be a successful conveyance to its terminus point. These superficial nodes are either steady at their starting points or they dynamically move in their planes. Let $S_{k}$ be the set of sinks in the network, then

(4) $$S_{k}=S_{k1},S_{k2},S_{k3},.\;.\;.\;.\;S_{kn}$$

where $S_{ki}$ is a specific node and n is the total number of sinks.

*Transmission Range ($T_{r}^{i}$) of Node(i)*: The transmission range of any node i($x_{s},y_{s},z_{s}$) is the omnidirectional distance up to where it can transmit the data packet.

*Eligible Neighbors of Node(i) ($EGN_{i}$)*: The nodes that are in the transmission range of Node (i) are the eligible neighbors of Node (i). This can be mathematically expressed as:

(5) $$EGN_{i}=i\epsilon S_{N}\wedge Dist_{i}^{j}\leq T_{r}^{j1}$$

where $S_{N}$ is the total numbers of nodes in the network and $Dist_{i}^{i}$ is the Euclidean distance between node $i(x_{i},y_{i},z_{i})$ and $j(x_{i},y_{i},z_{i})$.

(6) $$DIST_{j}^{i}=\sqrt{{\left(x_{i}-x_{j}\right)}^{2}+{\left(y_{i}-y_{j}\right)}^{2}+{\left(z_{i}-z_{j}\right)}^{2}}.$$

*Potential Forwarding Nodes (PFNs)*: The nodes that are in the set $EGN_{i}$ and lie in the upper hemisphere are called PFNs. The following condition must be satisfied for the node s1, which is the PFN of some other node s.

(7) $$PFNs\subseteq EGN_{s1}\wedge depth_{s1}<depth_{s}$$

*Potential Forwarding Zone (PFZ)*: PFZ is the bisection region or sub-region of the transmission range in which each point is nearer to the sink compared to the current forwarder and its radius is equal to the radius of the forwarder. The following conditions must be true for a point $ps(x_{ps},y_{ps},z_{ps})$ to be in PFZ of S in the 3D Euclidean space.

(8) $$Dist_{D}^{ps}<Dist_{D}^{S},Dist_{S}^{ps}<T_{r}^{S}$$

where

- $Dist_{D}^{ps}$ is the distance between point $ps(x_{ps},y_{ps},z_{ps})$ and Sink node $D(x_{D},y_{D},z_{D})$
- $Dist_{D}^{S}$ is the distance between point $S(x_{s},y_{s},z_{s})$ and Sink node $D(x_{D},y_{D},z_{D})$
- $Dist_{S}^{ps}$ is the distance between point $ps(x_{s},y_{s},z_{s})$ and Source node $S(x_{s},y_{s},z_{s})$.

## 4. Proposed Scheme

In this section, we present the details of our proposed scheme. The proposed protocol enhances the performance matrics of ESEVBF by extending the holding time mechanism of the first-hop forwarder to the second hop and further solves the hidden terminal problem.

### 4.1. Network Architecture

**Figure 4:**
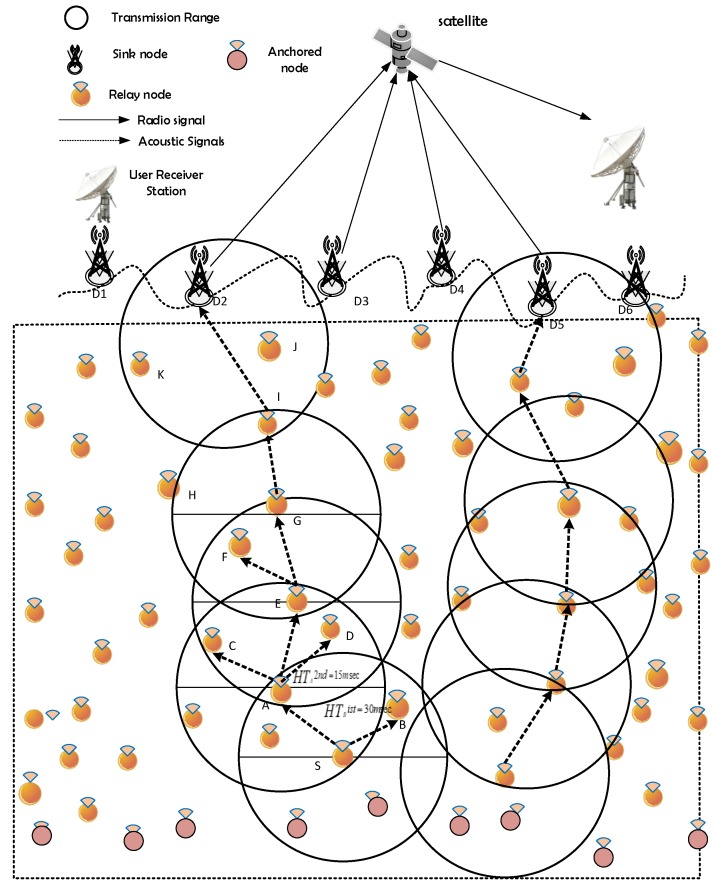
Network architecture.

The network architecture of EESEVBF protocol is composed of anchored nodes, relay nodes and sink nodes, as depicted in Figure 4. The terminus nodes/sink nodes are centralized stations consisting of acoustic and radio modems. They communicate with each other and with the external network through the radio links. Sink nodes are fixed at the water surface. The data received by any sink node is considered a successful delivery to its destination. On the other hand, relay nodes are movable with the water current while anchored nodes are fixed at the bottom. The sensor nodes communicate with each other through an acoustic link. The speed of acoustic signals (1500 m/s) is much smaller than that of electromagnetic signals (3.8 × 10${}^{8}$ m/s). Environmental monitoring and underwater tectonic plates monitoring are the typical applications of the network [33].

### 4.2. Acoustic Signal Velocity in the Underwater Environment

The speed of acoustic waves mainly depends on the variation of pressure, temperature, and salinity of water at different layers. Let us suppose that *C* represents acoustic sound velocity, *T* is the underwater temperature at different layers, *S* is the Salinity and *D* is used to represent depth, then mathematically, the speed of acoustic signal (C) can be expressed as [33]:

(9) $$\begin{array}{c} C=1448.96+\left(4.591\times T-5.304\times 10^{-2}\times T^{2}\right)+2.374\times 10^{-4}\times T^{3}+1.340\left(S-35\right)+1.63\times 10^{-2}D\\ +1.675\times 10^{-7}D^{2}-1.025\times 10^{-2}T\left(S-35\right)-7.139\times 10^{-13}\times D^{3} \end{array}$$

**Figure 5:**
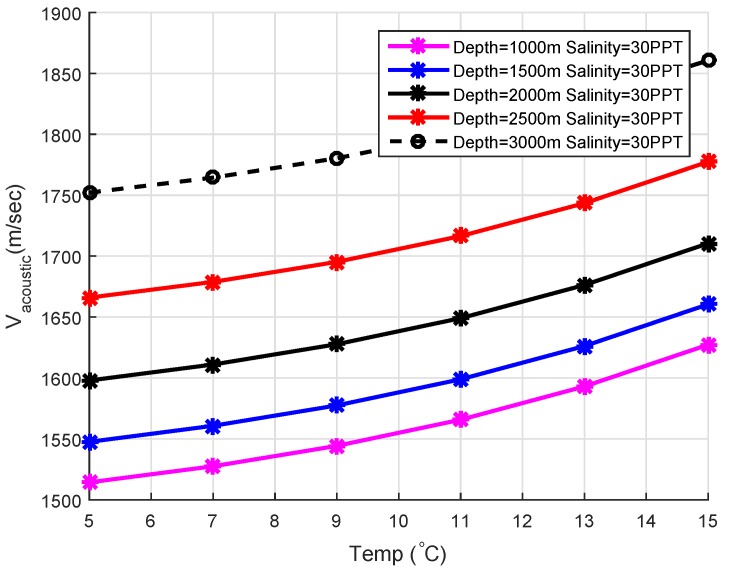
$V_{acoustic}$ vs. Temp.

**Figure 6:**
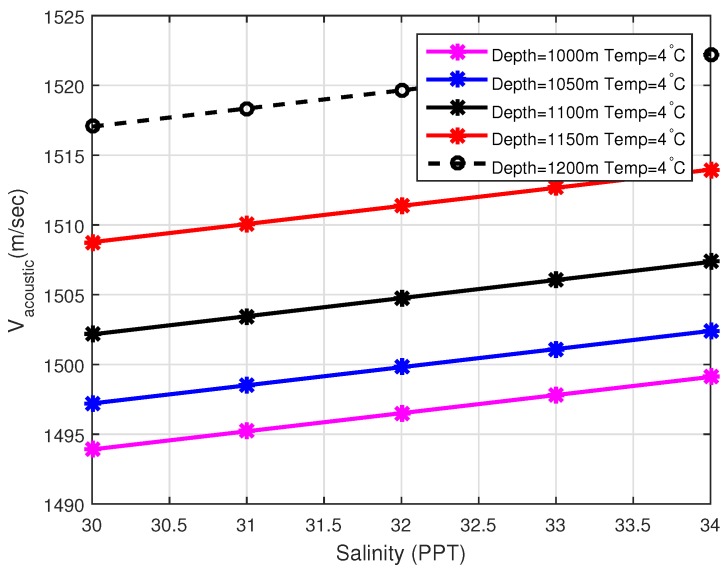
$V_{acoustic}$ vs. Salinity.

The above Equation is well grounded for 0 °C $\leq Temp\leq 30{\;}^{{}^{\circ}}$C, 30≤Si≤40PPT,0≤depth≤8000 m. The effects of temperature and salinity on acoustic sound velocity can be observed in Figure 5 and Figure 6.

### 4.3. Packet Types in EESEVBF

The proposed protocol defines five different types of packets, which are *Neighbor Request, Acknowledgment, Container, Announcement* and *Data Packet*. The sensor nodes are deployed in the 3D region. The holding time mechanism is used to select the next forwarder when the packet is broadcast by the source node and received by the PFNs. If the PFNs remain static throughout the simulation life, then one specific node will always be selected when the source broadcasts the packet. The selected node will go to a dead state in a limited time due to the repeated selection. Therefore, the sensor nodes move and randomly change their positions. The qualified forwarding nodes are shallower than the current source node and the distance between them is lesser than the transmission range. To find these forwarding nodes, the source node broadcasts the *Neighbor Request Packet*. The format of the *Neighbor Request Packet* is *NRP(id,dpt,ty)*, where *id* represents a unique integer number assigned to each node in the initialization phase, *dpt* contains information about the depth, and *ty* is a binary number used to differentiate between the packets. The value of *ty* for *NRP* is “00”. When the neighbor receives a *Neighbor Request Packet*, it replies with an *Acknowledgment Packet*. The format of an *Acknowledgment Packet* is *AP(id,dpt,ei,ty)*, where *id* is a unique number assigned to each node, *dpt* is the depth information, *ei* is the energy status and ty is “01” representing the packet type. *The Container Packet (CP)* is used to exchange the priorities at the first hop between the PFNs of the source node. The neighbor node, upon receiving a data packet from the source node, calculates its own holding time as well as finds the minimum holding time at the second hop. The format of a *Container Packet is CP(id,$HT_{1st}$,$HT_{2nd}$,ty)* where id is the unique number of the sending node, $HT_{1st}$ is the holding time of the node at the first hop, $HT_{2nd}$ is the sum of the holding time of the node at the first hop and with the minimum holding of the PFNs at the second hop. Similar to the other types, ty differentiates the packet with the value of “10” for CP. When the source node receives the Container packets from all the neighbors, it broadcasts the *Announcement Packet (AC)*. This type of packet is used for the solution of the hidden terminal problem discussed later in this section. The format of the *Announcement Packet* is AC ($hp_{id}$,x,y,z,ty) where $hp_{id}$,x,y, and z represents the id and coordinates of the highest priority node respectively and ty value for AC is “11”. The last packet type is *Data Packet (DP)*, which is the actual data to be sent to the centralized station. The format of *Data Packet* is *DP(HD,PD,ty)*. HD representing the header of the packet contains information about the packet generating node and the centralized station, PD is the payload of the data packet, and the ty value in this case is “111”. The payload is the most crucial part containing the actual information about the environment. If the above packets are transmitted in short intervals, then higher network overhead and energy consumption are expected. Therefore, to avoid this problem, each node keeps its corresponding neighbor table, which is for the purpose of keeping a track record of their neighbors. The format of the neighbor table is NeighTab(NID, Ei, Depth, $HT_{r}$, TU), where NID represents the neighbor node ID, Ei is the residual energy of the node, $HT_{r}$ is its holding time and TU represents the time for updating the neighbor entry. Meanwhile, if the source node initiates/receives the second packet when there is enough time for updating the neighbor table, then it will directly broadcast the *Data Packet* and the *Announcement Packet* from the previous holding time estimations.

### 4.4. Packet Forwarding Mechanism In EESEVBF

The proposed protocol extends the holding time mechanism of the first-hop forwarder to the second hop for finding the best satisfactory node with respect to the net packet advancement cover in the two hops. In addition, it also avoids the occurrence of a void hole whether it is due to an energy hole or the lack of potential forwarding nodes in the transmission range. There are two types of neighbors of a source node. PFNs are the nodes lying in the upper hemisphere of a source node, whereas suppressed nodes are the ones deeper than the source node. When a packet broadcast by the source node is received by a qualified node, it finds the energy of the neighbors from the neighbor table to calculate its holding time ($HT_{1st}$) at the first hop. In addition, the qualified node of the source node has sufficient information about expected PFNs at the second hop in its transmission range. The PFN further calculates its own holding time along with the expected nodes at the second hop. To find the $HT_{2nd}$ of the PFN, the holding time of the node with the highest priority among the second hop PFNs is added with its own holding time. There are two priorities of the qualified nodes, one at the first hop based on its own holding time, and the second is based on its own as well as the expected next PFN at the second hop. The nodes then exchange the container packet to know about the priorities of the other common PFNs with the source node.

**Figure 7:**
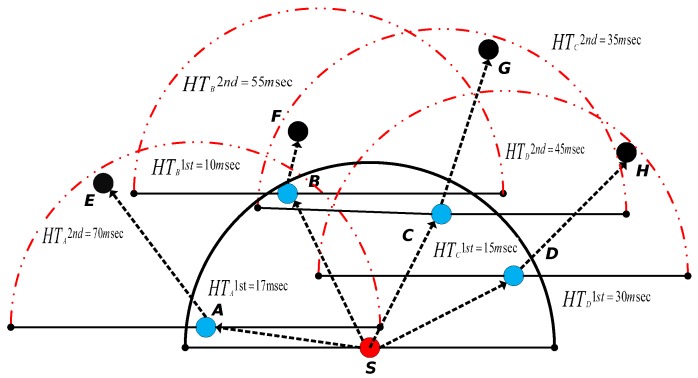
Forwarding Scenario.

The format of the sensor is $Struct(ID,HT_{1st},HT_{2nd},H_{Tr})$. The identifier ID is a unique integer number representing some specific node in the network and $HT_{1st}$, $HT_{2nd}$ represent the holding time values at the first and second hop respectively, while $HT_{r}$ is Resultant Holding Time/Timer value. The *Container Packet CP* received from the common qualified node contains the information about holding time/priorities of the sending node. Therefore, upon receiving the *CP*, the node places the values of ID, $HT_{1st}$, and $HT_{2nd}$ in Struct1. The node then sets its timer, i.e., HTr value after processing the priorities of all the forwarders. The overall holding time phenomena is the same for both first and second hop, only the holding time value is changed at the second hop to adjust the priority. At both first and second hops, the nodes with 1st priority sets their holding times $HT_{r}$. Similarly, the nodes with 2nd highest priority at each hop set their corresponding holding times and the same mechanism is applied to all the remaining nodes. Each node has its own structure and can calculate the holding times HTr for the remaining nodes as well. For instance, as shown in Figure 7, S is the source node with the PFNs A, B, C, D with IDs 1,2,3 and 4, respectively. Let node A after receiving the *Container Packet* from all the common PFNs with source has Struct1.ID (1,2,3,4), $Struct1.HT_{1st}\left(15msec,10msec,17msec,30msec\right)$ and $Struct1.HT_{2nd}\left(70msec,55msec,35msec,45msec\right)$, as listed in Table 1.

At the first hop, node B has its ID 2 and holding time value 10 msec, which is the lowest $HT_{1st}$ value and has the highest priority among all the nodes at the first hop. In contrast, node C with ID of 3 is at the 1st priority at the second hop node. Therefore, node C sets its holding time ($HT_{r}$) value as the holding time value of node B at the first hop. The second highest priority node D at second hop sets its holding time ($HT_{r}$) value with that of the holding of Node C at the first hop, similarly, node B with 3rd priority at the second hop sets it holding time value ($HT_{r}$) as the holding time of node A at the first hop. A similar mechanism is applied to all the remaining nodes. This results in the new struct1, as shown in Table 2. The nodes A, B, C and D then set their timer values to their corresponding $HT_{r}$ values. When the timer expires and no duplicate packet is received, the PFN forwards the packet. Algorithm 1 is the proposed algorithm for selecting the next PFN. The technical differences between the EESEVBF and ESEVBF lie in the mechanism used for holding time/timer value calculation. Our proposed protocol EESEVBF uses a novel approach in vector-based forwarding protocols by not disturbing the timer value in the first hop but only exchanging the priority of the transmission based on the subsequent hops.

Moreover, the proposed scheme uses *Announcement Packet* for the solution of the hidden terminal problem that occurs in ESEVBF. To understand this problem, consider the following scenario: A neighbor node receives a data packet from the source node. The qualified forwarding nodes calculate the holding times and set the timers accordingly. When the timer expires and the node does not receive the duplicate packet of the Data, it forwards the packet. The reproducing nodes are those that are in the range of the source node but not in the range of the node with the lowest holding time value among all the PFNs. The packet is forwarded by the node with the lowest holding time value, but the reproducing nodes do not receive a duplicate copy and hence, they also transmit the same packet. In contrast, our proposed scheme uses *Announcement Packet*, broadcast by the source node after receiving the *Container Packet*. The *Announcement Packet* contains the necessary information about the forwarder. When a node receives the *Announcement Packet*, it calculates its distances with the forwarder and checks if it lies in the range of the forwarder or not. If the statement is true, then it waits until the timer expires, otherwise it drops the packet. The reproducing nodes not lying in the range of the forwarder node drop the packet before the expiry of the timer. In this way, the generation of duplicate packets is suppressed resulting in lower energy consumption.
**Algorithm 1:** Proposed Algorithm for Selecting the Next PFN.
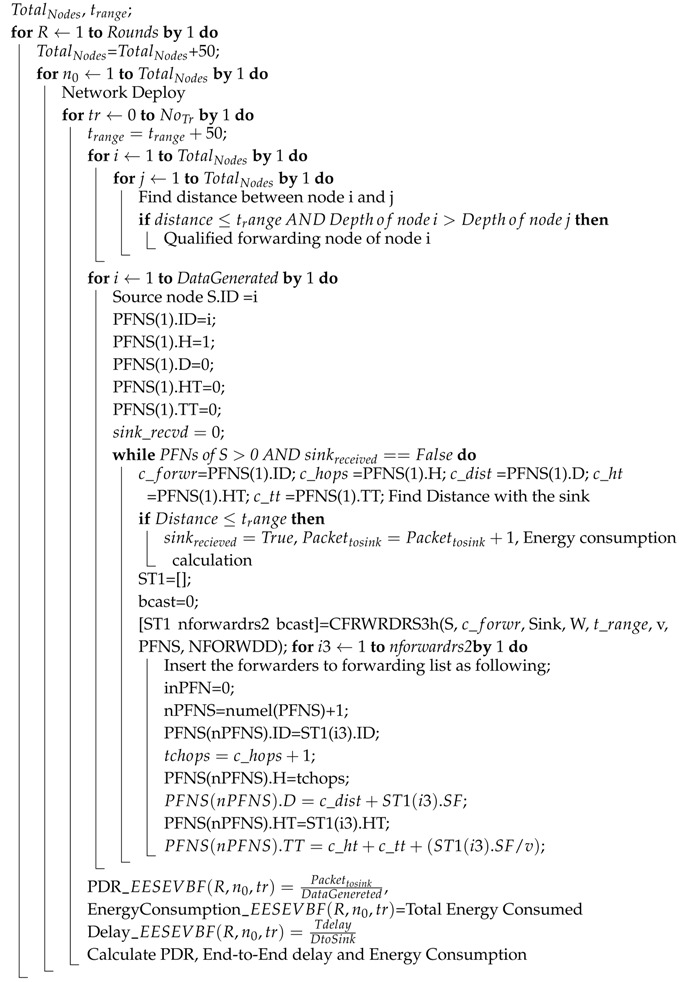


### 4.5. Holding Time Estimation

**Figure 8:**
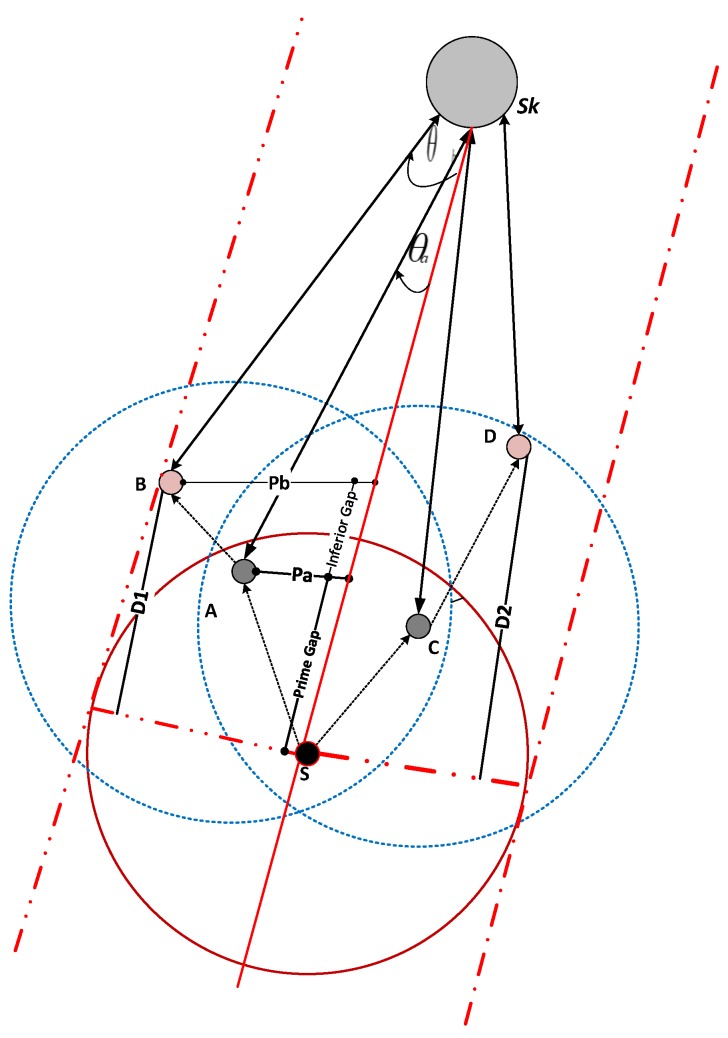
Holding time estimation.

The acoustic sensor nodes are deployed to monitor the underwater environment. The sensor nodes persistently originate data packets and forward them to the centralized station. When the source node broadcasts the data packets, all nodes lying in its transmission range receive them. If all the forwarder nodes take part in forwarding the packets, then it will result in the overhead and higher energy consumption. To increase the network lifetime, we propose a holding time mechanism (see Algorithm 2) to reduce the number of forwarding nodes to lower the energy consumption, but at the same time, ensure reliability. Once the required processing time is passed, the node extends the holding time value on the second hop by setting the timer according to the holding time value. In that case, the packet is forwarded only upon the timer’s expiry and no duplicate of the packet is received, otherwise, it is dropped. The following equations have been derived from [32] and processed further for the holding time mechanism of node A, as depicted in Figure 8.

(10) $$HT_{p}^{A}=E_{f}+P_{f}+CultivatePacketAdvancement$$

The parameter energy factor ($E_{f}$) represents the distance of the PFN from the edge of the transmission range scaled with the inverse normalized energy of the PFN at the first hop as well as at the second hop, i.e.,

(11) $$\begin{array}{cc} E_{f} & =e^{\left(-E_{A}\right)}\left(\frac{T_{r}^{S}-D_{S}^{A}}{v_{s}}\right) \end{array}$$

where

$$\begin{array}{cc} E_{A} & =\frac{\left(e_{A}+e_{B}\right)-\left(emin_{hp1}+emin_{hp2}\right)}{\left(emax_{hp1}+emax_{hp1}\right)-\left(emin_{hp1}+emin_{hp1}\right)}\\ e_{min} & =min\left(e_{j}|\forall j\in \chi_{i}\right)\\ e_{max} & =max\left(e_{j}|\forall j\in \chi_{i}\right)\\ E_{A} & \in [0,1] \end{array}$$

**Algorithm 2:** Estimation of the Holding time.

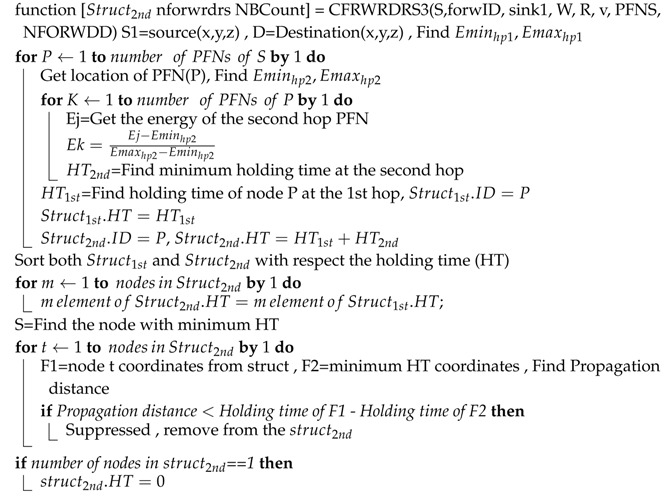



**Figure 9:**
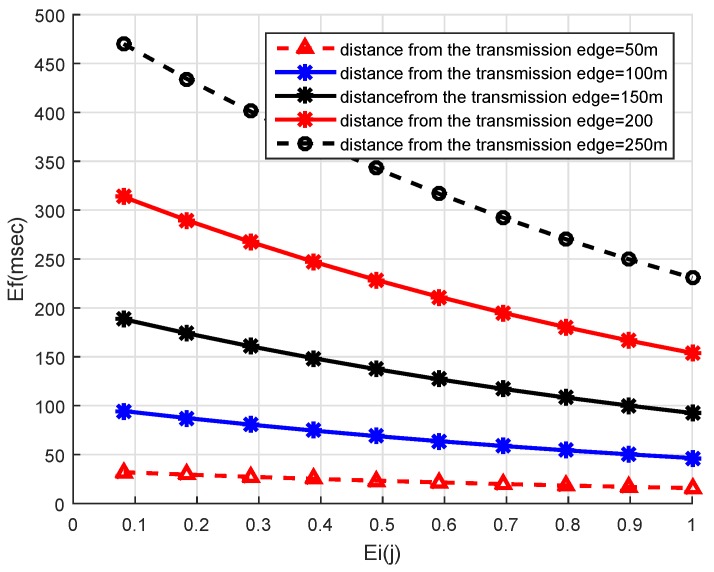
Ef vs. Normalized Energy.

The energy of the node is normalized in neighbors of the source node. The normalized energy is between 0 and 1. The node with maximum residual energy will have lower normalized energy value. The $e_{A}$ and $e_{B}$ represent the residual energy of nodes A and B, respectively. The $emin_{hp1}$ and $emin_{hp2}$ are the minimum energy of the PFN at the first and second hop respectively, the converse is true for $emax_{hp1}$ and $emax_{hp2}$. On the other hand, the normalized energy value is higher for the node which has higher residual energy for itself as well as for its expected PFN at the second hop than the specific threshold. Figure 9 is plotted by varying the distance of the PFN from the edge of the transmission range and its normalized energy. The $E_{f}$ factor decreases when the residual energy of the nodes increases and the distance from the edge of the transmission range decreases. The node with the highest residual energy among all the PFNs has a $E_{f}$ value approximately equal to zero.

The second factor $P_{f}$ in holding time calculation brings directionality and shows the placement of the node with respect to the virtual pipeline. The node nearer to the vector connecting sender and sink is preferable for forwarding the packets. Nodes that are not within the virtual pipeline directly drop the packet. The $P_{f}$ is calculated as:

(12) $$P_{f}=\tan\left(\frac{P_{a}}{W}\right)$$

$$\begin{array}{cc} P_{a} & =\left(2\times Z\right)/D_{Sk}^{S},\\ Z & =\sqrt{fr\times \left(fr-D_{Sk}^{S}\right)\times \left(fr{-}_{Sk}^{A}\right)\times \left(fr-D_{Sk}^{A}\right)},\;\mathrm{and}\\ fr & =\left(\frac{D_{Sk}^{S}+D_{Sk}^{A}+D_{Sk}^{A}}{2}\right).\; \end{array}$$

**Figure 10:**
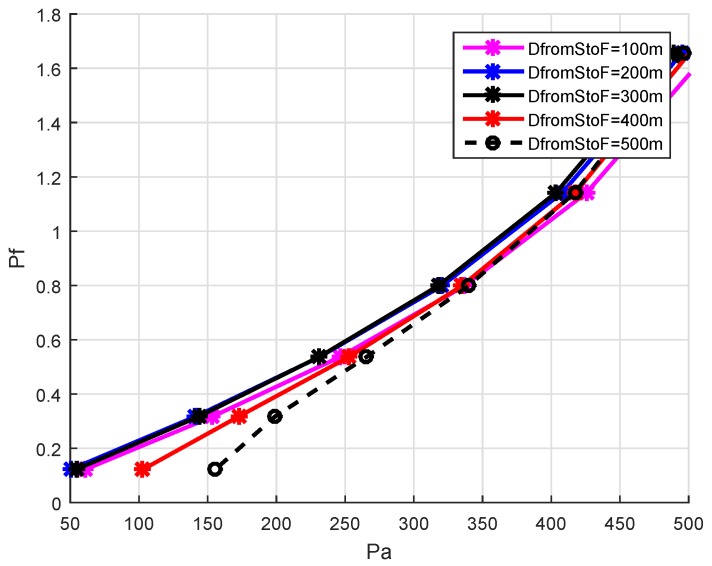
Forwarder Node Selection Scenario.

The $P_{f}$ increases when the distance $P_{a}$ from the virtual pipeline increases resulting in increasing the holding time of the forwarding node (see Figure 10).

(13) $$\begin{array}{c} CultivatePacketAdvancement=\overset{\delta }{\overset{︷}{2-(PrimeGap+inferierGap)}} \end{array}$$

The last factor *Cultivate Packet Advancement* represents the advance distance to be covered from the source node to PFN at the second hop of the forwarding packet. The factor *Cultivate Packet Advancement* of all the nodes is in the range [0,1].

(14) $$\begin{array}{c} CultivatePacketAdvancement=\overset{\delta }{\overset{︷}{2-\left(\frac{D_{Sk}^{S}-D_{Sk}^{A}\cos\left(\theta_{a}\right)}{T_{r}^{S}}\right)+\left(\frac{D_{Sk}^{A}-D_{Sk}^{B}\cos\left(\theta_{b}\right)}{T_{r}^{S}}\right)}} \end{array}$$

The nodes nearer to the sink have a lesser value of *Cultivate Packet Advancement*. The packet is advanced by the distance D1 from the source node when we select node A for forwarding. On the other hand, if node B is selected as a forwarder, the packet on the first hop is advanced by a lesser distance than node A. However, on the second hop, node B is more favorable. The factor $D_{S}^{B}k\cos\left(\theta_{a}\right)$ gives the distance from the sink node to the projection point of node A on the center of the pipeline. Similarly, $D_{S}^{B}k\cos\left(\theta_{j}\right)$ gives the distance up to the projection point of B on the center of the pipeline. Here, $\theta_{a}$ and $\theta_{b}$ are represented as:

(15) $$\theta_{a}=cos^{-1}\left(\frac{{D_{Sk}^{S}}^{2}+{D_{Sk}^{A}}^{2}+{D_{S}^{A}}^{2}}{2\times {D_{Sk}^{S}}^{2}\times D_{Sk}^{A}}\right)$$

(16) $$\theta_{b}=cos^{-1}\left(\frac{{D_{Sk}^{A}}^{2}+{D_{Sk}^{B}}^{2}+{D_{A}^{B}}^{2}}{2\times {D_{Sk}^{A}}^{2}\times D_{Sk}^{B}}\right)$$

Consider the nodes shown in Figure 8, ESEVBF will select node A for forwarding because it fulfills all the factors successfully as compared to node B at the first hop. However, Node B covers more advance distance from source node S to node D at the second hop. Therefore, the proposed system will select node C for forwarding the packet, resulting in lower end-to-end delay and energy consumption.

## 5. Experimental Setup

This section describes the details of our simulation setup, metrics, and evaluation methodology. In our simulations, we compare the performance of EESEVBF with ESEVBF. We deploy a 3D volumetric region of *length (2 km) × width (2 km) × depth (4 km)*. The header size of the data packet is 11 bytes, the payload is 72 bytes, neighbor request and acknowledgment are 50 bits, and the container packet size is 70 bits. The data rate is 16 kbps. The propagation speed of sound underwater is 1500 m/s. The number of nodes varies from 200 to 450 to demonstrate the performance in the sparse and dense networks, and the transmission range varies from 500 m to 900 m. The centralized station for receiving the data packets is static and at the water surface. The nodes can move with a constant speed of 2 m/s. The initial energy, sending energy, and receiving energy/idle energy of the node is set to 80J, 4.5J, and 0.017J, respectively. The background underwater wireless environment and the acoustic channel parameters (ambient noise, site-specific noise, central acoustic signal frequency) are set for simulation, as given in [27].

## 6. Analysis of Results

The proposed protocol intensifies the performance parameters (end-to-end delay, PDR, energy consumption, and duplicate packets) of ESEVBF. The underwater network is deployed to monitor some specific areas in the sea roof.

### 6.1. Packet Delivery Ratio (PDR)

The source nodes consistently generate the data packets and forward them to the destination. PDR is the ratio between the number of packets received at the destination to the packets created and sent by the source node. There can be more than one copy of a particular packet received, but we only consider one at the destination. The PDR is calculated as follows:

(17) $$PDR=\frac{Packets\;received\;at\;the\;centralized\;station}{Packets\;sent\;by\;the\;source\;node}$$

**Figure 11:**
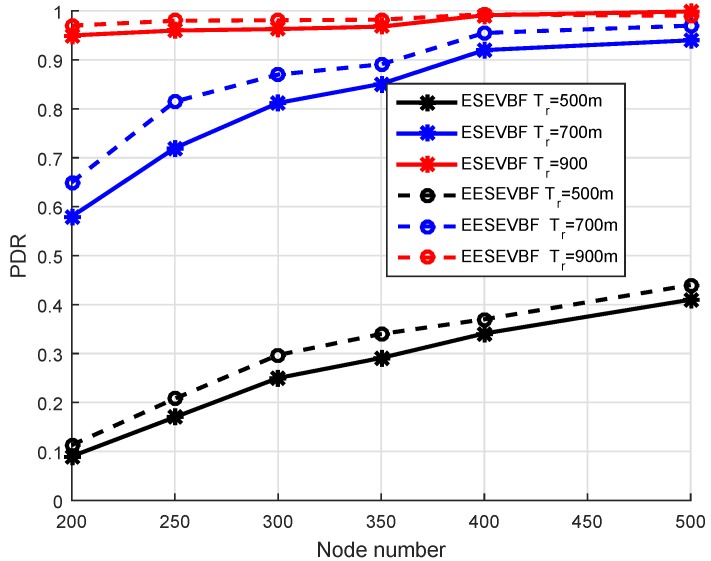
Number of Nodes vs. PDR.

In Figure 11, the simulation results of PDR are plotted by varying the number of nodes and the number of transmission ranges. For both protocols, the PDR increases when the number of nodes increases. It satisfies the fact that when the nodes increase, then in the transmission range of each node, there will be more numbers of nodes, which causes a reduction in the packet drops and increases the reliability. The probability of a void hole occurrence is reduced when the network changes from the sparse towards a denser one. As evident from Figure 11, the PDR of EESEVBF is greater than that of the ESEVBF because the former considers the second hop PFN while selecting the PFN at the first hop. There are two possibilities of void hole occurrence, one when there is no PFN and the second when there is PFN in the range of the source node, but it does not have sufficient energy to forward the packet. The proposed protocol avoids both cases by considering the holding time of the PFN at the second hop.

**Figure 12:**
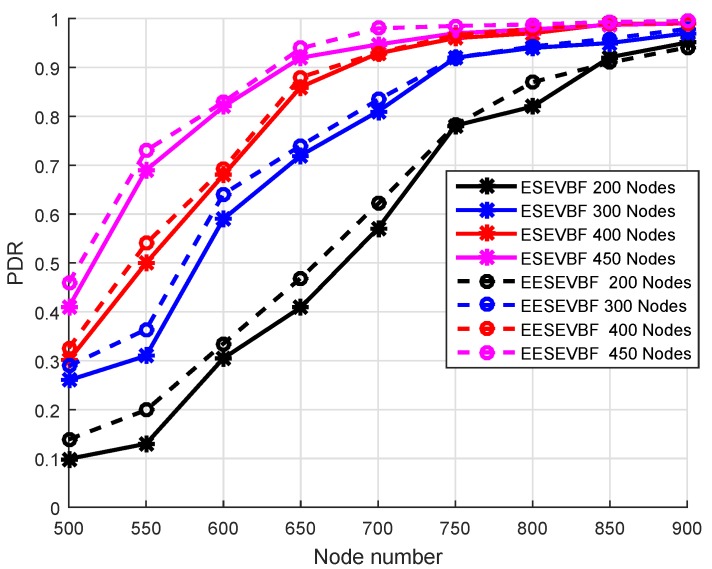
Number of Nodes vs. PDR.

For the transmission range of 500 m, the PDR of EESEVBF is slightly higher than ESEVBF; however, the difference is small compared to the 700 m transmission range, as shown in Figure 11. This is because, for small transmission range, the holding time of the proposed protocol is not effective compared to the large transmission, as shown in Equation (10). In this case, the selection of the node is purely based on the first hop as in ESEVBF; however, for large transmission range, the selection is prioritized from the combination of the first and the second hop. When the transmission range increases for the same number of nodes, the PDR is also increased. In this case, there is an increased probability of finding a more suitable node with respect to the holding time; however, for lower transmission range, it is difficult to find the next PFN and the packet cannot reach even at the fourth hop from the source node. Similarly, as shown in Figure 12, the difference between PDR of the two protocols is high when the number of nodes is 200 and the transmission range increases compared to the denser ones. The reason is that in a dense network, there are sufficient nodes in the transmission range for selecting the PFN, and therefore, the next forwarder of the PFN gets a negligible effect on the PDR. The converse is true for sparse networks.

### 6.2. Energy Consumption

**Figure 13:**
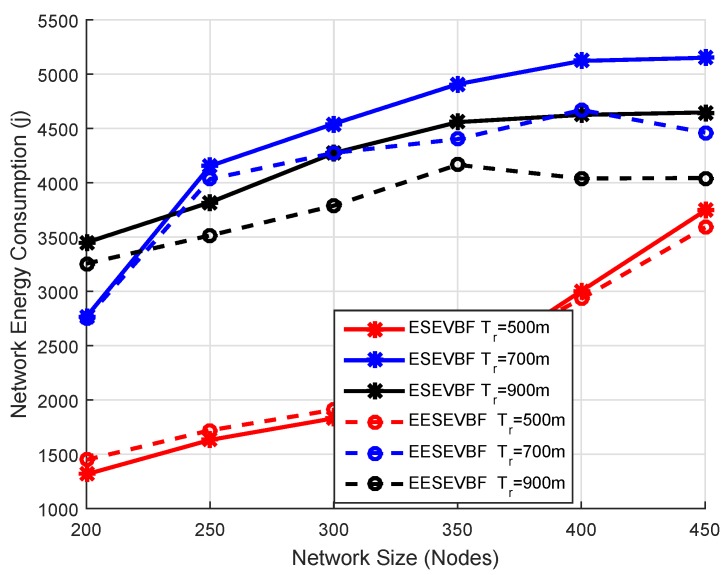
Number of Nodes vs. Energy Consumption.

**Figure 14:**
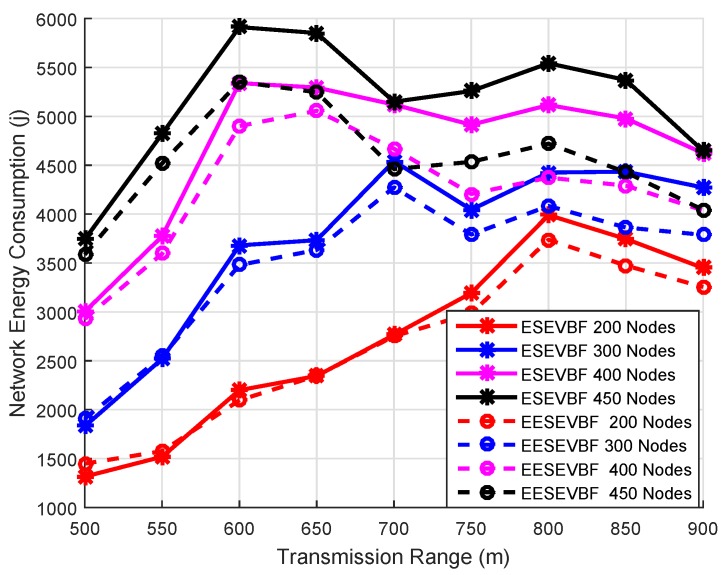
Number of Nodes vs. Energy Consumption.

**Figure 15:**
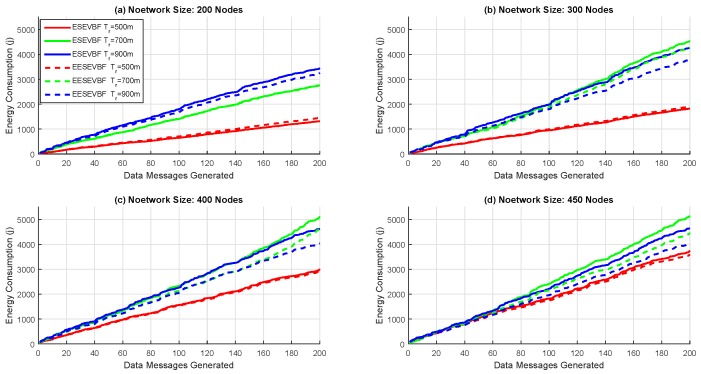
Number of Nodes vs. Energy Consumption.

**Figure 16:**
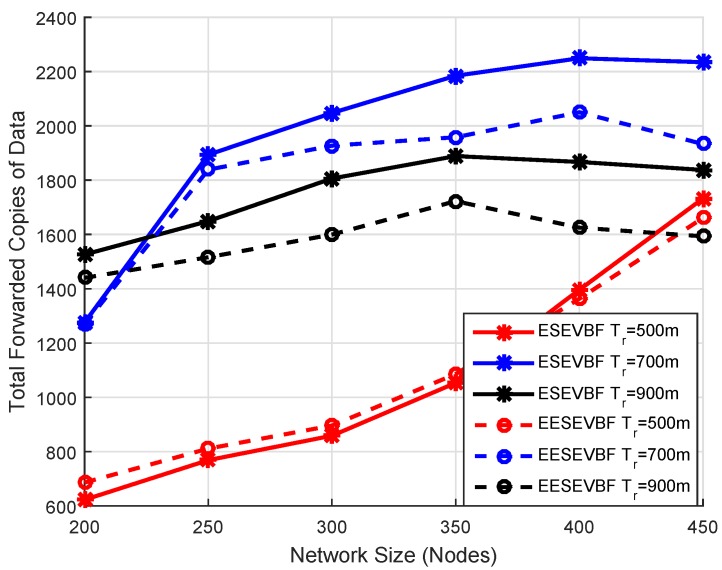
Number of Nodes vs. Data Copies Forwarded.

**Figure 17:**
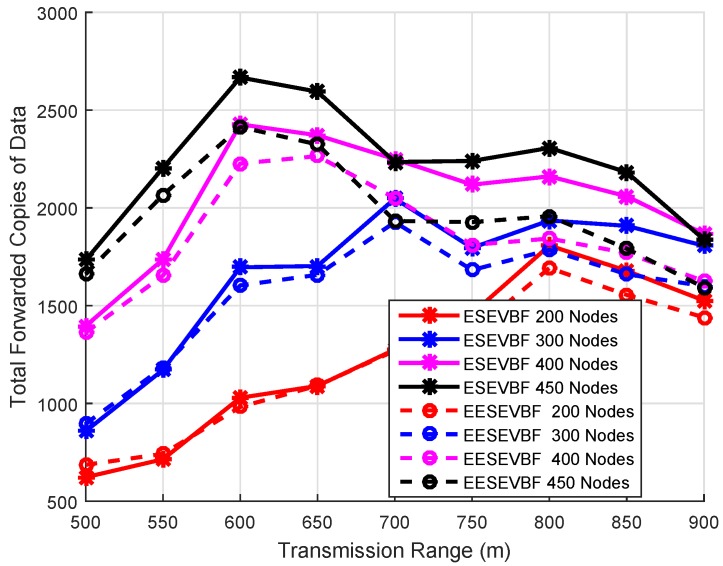
Number of Nodes vs. Data Copy Forwarded.

**Figure 18:**
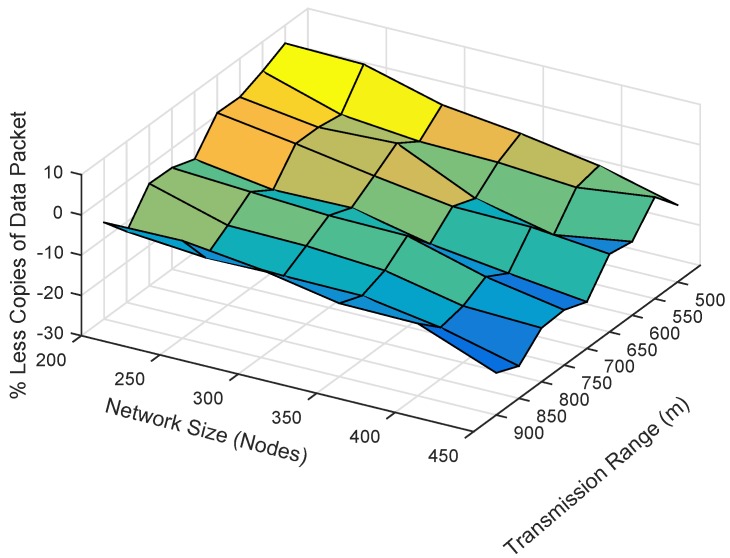
Number of Nodes vs. Data Copy Forwarded.

Energy consumption is the total amount of energy consumed in the network throughout the whole simulation. It is known that the energy consumption increases when the number of nodes increases, because more duplicate packets are generated and forwarded. We plot the simulation results of energy consumption by varying the number of nodes and the number of transmission ranges. The energy consumption of EESEVBF is lower than that of ESEVBF, as shown in Figure 13, Figure 14 and Figure 15. The reason is the instantiation of the duplicate packets. In ESEEVBF, the reproducing nodes generate the duplicate copies and forward them to their PFNs, as discussed in Section 4. The transmission energy on the duplicate packet is consumed from the reproducing node to the sink, which results in increased total energy consumption. In contrast, our proposed protocol not only selects the favorable path with respect to the packet advancement and residual energy at the first and second hop, but also tackles the hidden terminal problem (as discussed in Section 4) resulting in a reasonable reduction in duplicates packets, as shown in Figure 16, Figure 17 and Figure 18.

The energy consumption increases when the number of nodes increases. This is because some of the packets do not reach their fourth hop in the sparse network, resulting in lower packet delivery ratio and energy consumption. On the contrary, most of the packets are successfully delivered causing higher energy consumption. The difference between EESEVBF and ESEVBF is lessened for the lower number of transmission range and nodes because of the next forwarder selection mechanism. From Equation (1), it is difficult to find the node at the second hop, which competes with the first-hop forwarder nodes in case of a sparse network, and the forwarder is purely selected from the weight of the first-hop PFNs. Therefore, in large transmission ranges and dense networks, the effectiveness of our proposed protocol is clearer. The simulation results show that the proposed protocol reduces the energy consumption by an average of 10.45% in comparison to ESEVBF, as listed in comparison Table 3.

### 6.3. Energy Optimization

Linear programming is used when prime resource allocation is required. Linear programming is defined under some constraints in the linear objective function. The network performance and lifetime of any routing protocol can be enhanced by considering the energy consumption. To reduce the energy consumption, the proposed protocol also uses linear programming techniques. Energy tax minimization can be achieved as:

(18) $$minimum\sum_{r=1}^{r_{max}}Etax^{n}\left(r\right)\;\forall \;r\;\epsilon r_{max}$$

Energy tax is the energy consumed by each node per packet upon the successful delivery to its terminus point. It depends on the total energy consumed by the network, the number of nodes, and the total packets received by the destination node. The mathematical expression of energy tax can be written as:

(19) $$E_{tax}=\sum_{n=1}^{N}\frac{Etotal}{n*Packet\_to\_sink}$$

#### 6.3.1. Constraints

(20) E_node≤E_initial

(21) $$Forwarder\left(HT_{r}^{n}\right)\leq MiniForwarder\left(minHTr_{n=1}^{N}\right)$$

(22) $$Tr_{n}\leq Tr_{max}\;\forall \;n\;\epsilon \;N$$

The purpose of the objective function is to reduce total energy consumption. One of the constraints is that the energy provided to each node should be less than the initial energy as stated in Equation (20). The second constraint is that the forwarder of each source node is selected on the value of holding time and is designed in such a way that the node with maximum residual energy will have higher chances of selection. The last constraint is that the transmission range of each node should be less than the maximum required transmission range.

#### 6.3.2. Graphical Analysis

Suppose that $Nodes_{max}=450$, $Tr_{min}^{n}=500$, $Tr_{max}^{n}=900$, $E_{initial}=50j$, $E_{idle}=158$ mW, $Header_{Size}=11$ bits, *Payload* = 72 bits, $Dist_{S}^{F}=500$ m, $E_{normalized}=0.7j$, so the below equations are obtained from the above constraints.

(23) $$7.5\leq Etr^{n}\leq 11.11$$

(24) $$1.58\leq Ercv^{n}\leq 3.16$$

(25) $$9.08\leq Etr^{n}+Ercv^{n}\leq 14.27$$

**Figure 19:**
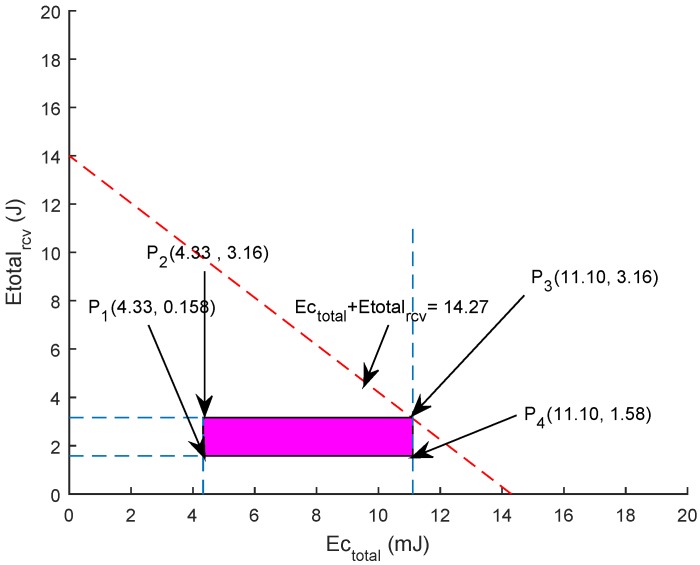
Feasible Region.

Equations (23)–(25) are drawn to find the feasible region, as shown in Figure 19. The optimal solution is validated by each vertex of the feasible region. The boundary points of the feasible region recognize the optimal solution. The points are: P1 (7.5, 1.58) = 9.08 J; P2 (7.5, 3.16) = 10.6 J; P3 (11.11, 1.58) = 12.69 J; P4 (11.11, 3.16) = 14.27 J.

The bounded region in Figure 19 indicates that the formulation is valid and energy usage in that specified region will always result in optimal network lifespan.

### 6.4. End-To-End Delay

**Figure 20:**
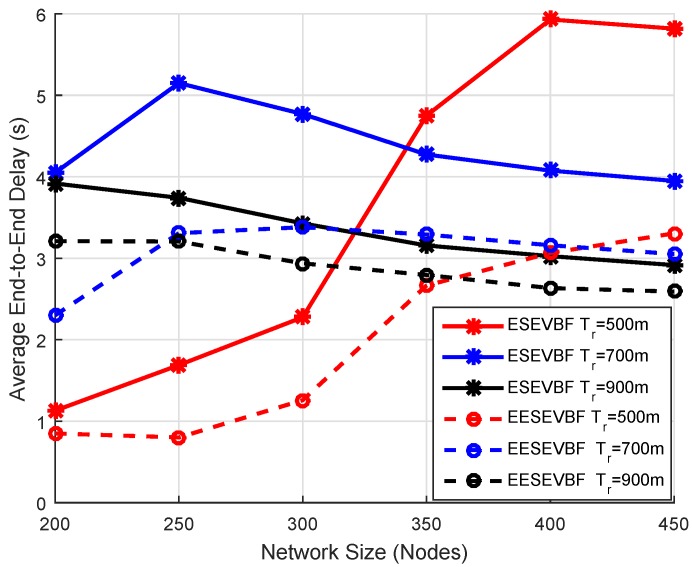
Number of Nodes vs. End-to-End delay.

**Figure 21:**
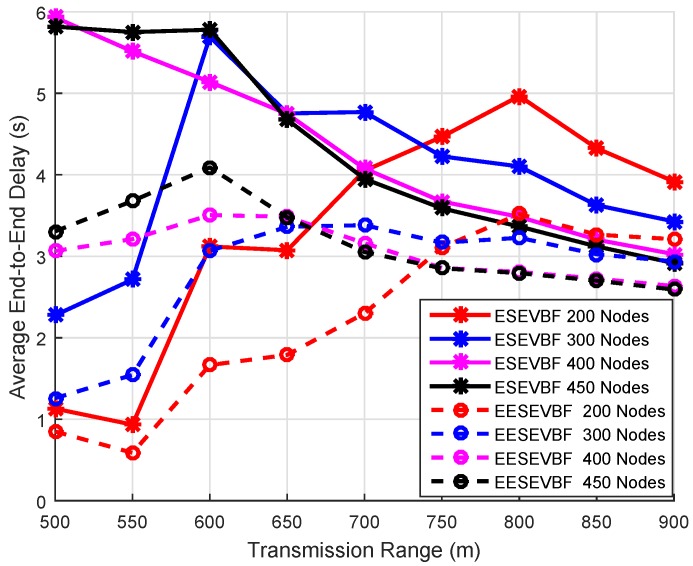
Number of Nodes vs. End-to-End delay.

**Figure 22:**
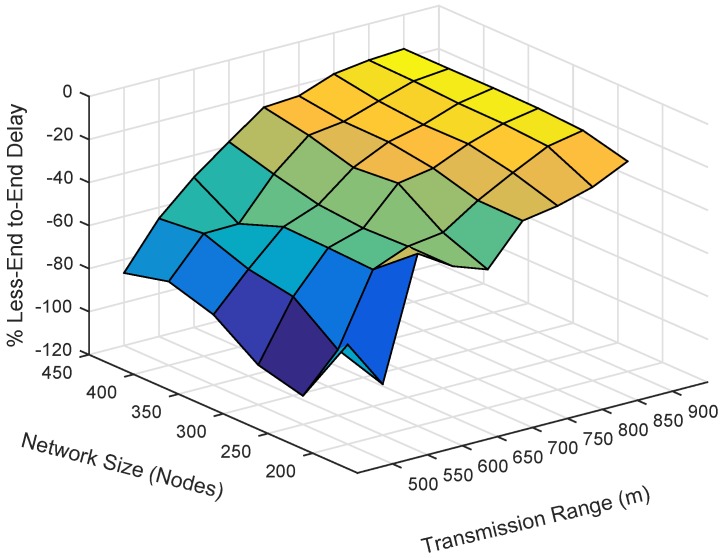
Number of Nodes vs. End-to-End delay.

End-to-End delay is the time taken by a packet in traversing a distance from the source node to the centralized station. It includes transmission delay, propagation delay, and the necessary processing time for the sensor node to forward a packet. The result of the end-to-end delay is plotted by varying the number of nodes and the transmission range. The end-to-end delay of EESEVBF is lower than that of ESEVBF, as shown in Table 4 and Figure 20, Figure 21 and Figure 22. There are two reasons for this:

- When a node forwards a packet, the next forwarder is selected based on the holding time algorithm. ESEVBF protocol only considers the advance distance covered in the 1st hop from the source to the forwarder in selecting the next PFN and not the one in the second hop of the respective PFN. On the other hand, the proposed protocol extends the holding time up to the second hop of the source node, i.e., the holding time mechanism of the forwarder node includes the distance from the edge of the transmission range of the source node to the forwarder, the distance of the source node to the second hop forwarder, the distance between the sink and next forwarder, and the distance from the virtual pipeline.
- The EESEVBF uses a container packet in which the nodes exchange their holding times at both the first and second hops. The nodes found from the holding time of each other set their priorities at the first and second hop, then only the one with the highest priority at the second hop transmits the packet within no time.

### 6.5. Analysis of Duplicate Packets

The total number of duplicate packets forwarded in the network is plotted by varying the transmission range and the network size, as shown in Figure 16 and Figure 17. The total forwarded copies in the sparse network are lesser than those in the denser network, as shown in Figure 16. The reason is that most of the packets do not reach their destinations and are dropped earlier from traversing even four hops from the source node. For bigger networks and transmission range, a large number of duplicate packets is forwarded in the network due to the reason that the pipeline radius increases and the number of PFNs in the potential forwarding zone also increases. The second reason is that the propagation delay between the two forwarders is greater than the holding time difference.

The number of copies forwarded in ESEVBF is greater than that in EESEVBF, which is due to the hidden terminal problem, as discussed in Section 3. Although the holding time is expanded in ESEVBF in such a way that the holding time difference between the two forwarder nodes is greater than the propagation delay between them; however, due to the hidden terminal problem, the reproducing nodes initiate duplicate packets. The proposed protocol suppresses the instantiation of the duplicate packets from the reproducing nodes by using *Container* and *Announcement Packets*. For the transmission range of 900 m, it forwards, on average, 13.5 lesser copies than the ESEVBF. For lower transmission range and small network size, ESEVBF performs better in terms of forwarding copies than the EESEVBF (see Figure 16). The reason is that the source node has the very least number of forwarding nodes in the transmission zone, and according to the holding mechanism of ESEVBF, the holding time difference is greater than the propagation delay between them, which causes the suppression of more data packets. In this case, the proposed protocol is not much effective due to the small number of nodes and the higher probability that all nodes are in the transmission range of one another, thus overcoming the occurrence of the hidden terminal problem. In contrast, for higher transmission, by solving the hidden terminal problem, the proposed protocol suppresses duplicate packets more than those by ESEVBF. The similar trends can be seen from Figure 17, when the transmission range increases for the same number of nodes, the difference between the forwarded copies of the two protocols is also increased. The effectiveness of the proposed protocol increases as the transmission range increases and the reason is clear, i.e., the suppression of duplicate packets initiated by the reproducing nodes.

## 7. Conclusions

In Underwater Wireless Sensors Networks (UWSNs), the sensor nodes are sparsely deployed due to deployment and high manufacturing cost in large deployment regions. To improve the reliability and reduce the energy consumption, we proposed a reliable and energy-efficient Extended Energy-Scaled and Expanded Vector-Based Forwarding Protocol (EESEVBF). The existing ESEVBF protocol fails when there is a void or energy hole at the second hop, which decreases the reliability of the system. The proposed protocol extends the holding time mechanism of the first-hop forwarder to the second hop for finding the best satisfactory path. EESEVBF overcomes the occurrence of void hole, whether it occurs to an energy hole or lack of a potential forwarding node in the transmission range. Moreover, the proposed protocol also tackles the hidden terminal problem due to which a reasonable reduction occurs in duplicate packets initiated by the reproducing nodes. The simulation results show that EESEVBF is 6.66% (approximately) more energy-efficient, experiences 20.2% less delay, and generates about 11.26% fewer data packets, compared to ESEVBF. Our experimental results demonstrate the efficiency of our proposed EESEVBF protocol over ESEVBF. The only overhead of our approach is the added complexity of computation in timer values, i.e., for a single routing decision, the system needs to exchange several control packets to estimate the timer values for all nodes up to the second hop in the locality of the source node. However, such calculations do not come with a high cost, i.e., the size of control packets range from 50–70 bits, which consumes negligible bandwidth and energy compared to the data packets.

In our current work, sensor nodes are deployed at random locations in the underwater network. One of the future works is to deploy sensor nodes at such locations that can balance the energy consumption. This may be possible by using some statistical distribution (e.g., Gaussian distribution) for the deployment of sensor nodes with respect to the virtual pipeline and sink nodes. In addition, the use of radio frequencies can also be investigated in our proposed EESEVBF protocol in future.

## Figures and Tables

**Table 1:** Packet Forwarding Scenario before the nodes receive the CP Packet.

Struct1	Node A	Node B	Node C	Node D
ID	1	2	3	4
$HT_{1st}$	17 msec	10 msec	15 msec	30 msec
$HT_{2nd}$	70 msec	55 msec	35 msec	45 msec
$HT_{r}$	—	—	—	—

**Table 2:** Packet Forwarding Scenario after the nodes received the CP Packet.

Struct1	Node A	Node B	Node C	Node D
ID	1	2	3	4
$HT_{1st}$	17 msec	10 msec	15 msec	30 msec
$HT_{2nd}$	70 msec	55 msec	35 msec	45 msec
$HT_{r}$	30 msec	17 msec	10 msec	15 msec

**Table 3:** Overall Energy Consumption improvement of EESEVBF compared to ESEVBF.

*Network Size*	300	400	450
$T_{r}=500$ m	0.81	1.16	2.50
550 m	0.10	2.53	5.32
600 m	2.55	7.62	10.21
650 m	1.31	5.32	10.90
700 m	4.16	8.33	11.12
750 m	4.50	12.51	13.33
800 m	6.96	11.78	14.16
850 m	10.8	10.83	15.81
900 m	9.16	8.33	10.90
**% Improvement**	**4.75%**	**7.59%**	**10.45%**

**Table 4:** Overall end-to-end delay improvement of EESEVBF compared to ESEVBF.

*Network Size*	200	300	400	450
$T_{r}=500m$	4.52	16.3	50.1	45.9
550 m	4.43	18.3	37.9	35.5
600 m	21.2	43.3	27.1	30.7
650 m	20.3	26.6	24.4	23.2
700 m	30.2	25.1	15.4	11.3
750 m	21.1	16.7	15.9	11.6
800 m	23.3	12.4	9.50	7.39
850 m	18.2	6.34	5.53	4.90
900 m	12.2	5.80	4.08	4.44
**% Improvement**	**17.1%**	**18.7%**	**20.2%**	**19.5%**
